# Progressive Deformity of the Lower Limbs in a Patient with KID (Keratitis-Ichthyosis-Deafness) Syndrome

**DOI:** 10.1155/2020/8747392

**Published:** 2020-07-10

**Authors:** Oleg Kozhevnikov, Svetlana Kralina, Yulia Yurasova, Vladimir Kenis, Susanne Gerit Kircher, Ali Al Kaissi

**Affiliations:** ^1^Orthopedic Children's Department, Central Research Institute of Traumatology and Orthopedics n.a. N.N.Priorov, Moscow, Russia; ^2^Department of Hospital Pediatrics No. Two of Russian National Research Medical University, Moscow, Russia; ^3^Department of Foot and Ankle Surgery, Neuroorthopaedics and Systemic Disorders, Pediatric Orthopedic Institute n.a. H.Turner, Saint Petersburg, Russia; ^4^Department of Medical Chemistry, Medical University of Vienna, Vienna 1090, Austria; ^5^Ludwig Boltzmann Institute of Osteology at Hanusch Hospital of OEGK and AUVA Trauma Centre Meidling, 1st Med. Dept. Hanusch Hospital, Vienna, Austria; ^6^Paediatric Department, Orthopaedic Hospital of Speising, Vienna, Austria

## Abstract

**Purpose:**

Progressive deformity of the lower limbs can be encountered in a long list of syndromic associations. The baseline tool in the management of such disorders is to approach to a definite diagnosis.

**Methods:**

We describe a 4-year-old girl who presented with the clinical phenotype and genotype of congenital erythrokeratoderma, keratosis, and sensorineural hearing loss (keratitis-ichthyosis-deafness syndrome) (KID syndrome). She manifested progressive contractures of the knees associated with talipes equinovarus of the feet. The latter deformities were the main reasons behind her severe retardation in acquiring the normal locomotor functions.

**Results:**

The analysis revealed mutations in intron 1 of the GJB2 gene of C.32G>A (p.Gly11Glu) and c.35delG in the compound heterozygous state. The presence in the genotype of the “dominant” mutation c.32G>A (p.Glu11Glu) was compatible with the clinical phenotype of KID syndrome.

**Conclusion:**

Surgical interventions through the extension of the hamstring tendons have been performed successfully via the application of an external distraction apparatus, namely, Volkov- Oganesyan. The latter procedures resulted in total release of her awkward knee contractures. Eventually, the child was able to regain the physiological alignment of her lower limbs and resume walking. To the best of our knowledge, the overall management of this child could be the first in the literature.

## 1. Introduction

Keratitis-ichthyosis-deafness (KID) syndrome is a rare form of ectodermal dysplasia caused by certain mutations in the GJB2 gene (connexin 26 gene) with characteristic lesions of the skin, hair, and hearing. For the first time, generalized congenital erythroderma was described by Burns in 1915 [1]. Skinner et al. in 1981 gave the name of the syndrome—keratitis-ichthyosis-deafness (KID) syndrome [2]. Congenital erythrokeratoderma, keratosis, and sensorineural hearing loss are the essence of the diagnosis. The skin is characterized by congenital erythrokeratoderma, which involves the cheeks; nose; chin; ears; limbs, especially the extensor and flexor surfaces of the elbows and joint areas of the knees, hands, and feet; and the buttocks. In some cases, scalp involvement results in partial or complete alopecia. The nails are frequently thickened and dystrophic. The palmar and plantar areas usually show hyperkeratosis characterized by a stippled or dotted pattern [3–5]. The nails are variably affected, and the hair is scanty and brittle. Hair histology might resemble that seen in trichothiodystrophy with bright and dark bands. Sweating is usually decreased. The deafness is congenital and of a sensorineural type, and a vascularized keratitis develops which is always preceded by photophobia. Many patients have a troublesome tendency to develop candida infections, supposedly because of an immune deficiency [6].

Mutations in the gene GJB6 (Cx30) also implicated in the Clouston syndrome have been found in KID with congenital atrichia [7], though Clouston syndrome is composed of a different clinical phenotype (of a hidrotic ectodermal dysplasia) characterized by fine, depigmented hair which is sparse on the head, eyebrows, and body. In addition, the nails are dystrophic, and there is often dyskeratotic skin on the palms and soles, which cracks easily and is rough to the touch. The skin over the knuckles, elbows, knees, axillae, and pubic areas shows hyperpigmentation and looks clinically like acanthosis nigricans. An X-ray of the skull might show thickening of the skull bones, and there might be tufting of the terminal phalanges [8].

Others reported that missense mutations in GJB2 encoding connexin 26 cause the ectodermal dysplasia keratitis-ichthyosis-deafness syndrome [8].

In our current child, spasticity of the hamstrings was the reason behind the progressive knee flexion deformity. In practice, knee contractures if left untreated lead to increasing load across the patellofemoral joint, resulting in pain and late arthritis. A decrease in the dynamic range of gait is common secondary to spasticity of the rectus femoris. Distraction of the lower extremity with an external fixator, to preserve the potential of the joint to restore its functional potential, was mandatory. Therefore, we referred to realignment procedures of the lower limbs for our KID syndromic child by means of a Volkov- Oganesyan apparatus to resume the functional restoration of joint deformities [9–11].

## 2. Clinical Report

A 4-year-old girl was referred to our department because of progressive deceleration in her motor skills. Deformities of the lower extremities ended up with contractures over her knees and feet. She was delivered on time, a product of 38-week gestation for unrelated parents. Family history was noncontributory. Immediately after her birth, skin changes of dryness and peeling were noted. Her early motor development was relatively normal, she began to sit at 8 months, stood up with support on her feet from 10 months, began to walk independently at the age of 14 months. At the age of 18 months, she was noted of being unable to extend her lower legs. The latter occurred because of progressive flexor contractures which left the child totally crippled. At the age of 3 years, she underwent cochlear implantation (in a deaf clinic). Simultaneously, a condensed treatment program for her skin lesions was carried out by a dermatologist. Clinical examination at the age of 4 years revealed postnatal proportionate growth deficiency. The patient's whole body skin was diffusely thickened showing follicular keratosis with erythema of the entire surface of the skin, more pronounced on the head and torso. Ichthyosis-like skin changes were characterized by dryness, thin scales, peeling, and thickening of the skin in the perioral zone. The mucous membrane of the mouth is also affected, with leukoplakia associated with fungal white plaque on the tongue and gums. Similarly, on the mucous surface of the inner part of the lips is a noticeable white growth confused with papilloma. There is no hair on the whole body, alopecia totalis, and no eyebrows and eyelashes. Extensive palmoplantar hyperkeratosis and hypoplatic nails are additional prominent features.

**Figure 1:**
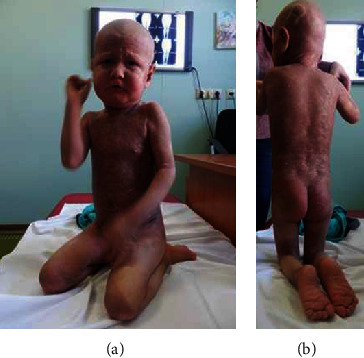
(a, b) The girl at admission; she manifested extensive lesions of the whole body (hyperkeratosis, erythroderma, alopecia totalis, lack of eyebrows and eyelashes, and characteristic wrinkled forehead) (a). Musculoskeletal examination showed fixed flexion contracture of the knees. At this stage, the child was unable to extend the knees and she could only crawl (b).

Distraction test via her mother's voice was positive. Musculoskeletal examination showed fixed flexion contracture of the knees. At this stage, the child was unable to extend the knees and she could only crawl (Figures 1(a) and 1(b)). We assessed the flexion deformity of the knees through studying the active and passive range of the hip, knee, ankle, and foot, in order to determine at which point the spastic muscles grab. The patient had a difficulty sitting on the floor with the knees in extension. She has to flex her knees to diminish the pull of the hamstrings on the ischium and pelvis. In patients with moderate hamstring contracture, when they sit, their lumbar spines assume a kyphotic posture. Our current patient manifested severe contracture of the hamstring; she tends to slide down the chair and has to be strapped down with the knees in flexion.

On the bases of skeletal survey, AP pelvis radiograph showed coxa valga associated with defective ossification of os pubis. Lateral knee radiograph showed fixed flexion deformity of the knee associated with small patella, and the foot showed equinus deformity.

### 2.1. Molecular Genetics

DNA was studied to search for mutations in the gene GJB2 encoding the protein connexin 26. The whole coding region of the GJB2 gene (exon 2) was sequenced by PDRF analysis of splicing site mutation c.-23+lG>A (IVSl+lG>A) in intron 1 of the GJB2 gene. The analysis revealed mutations of c.32G>A (p.Gly11Glu) and c.35delG in the compound heterozygous state.

In this study, we identified GJB2-associated hearing loss with palmoplantar keratoderma caused by compound heterozygous autosomal dominant c.32G>A (p.G11E) and autosomal recessive *GJB2* gene c.35delG (p.Gly12Valfs∗2) mutations. The combination of autosomal dominant syndromic hearing loss *GJB2* mutation with an autosomal recessive nonsyndromic hearing loss *GJB2* mutation is quite rare and limited in number of reports to date.

The c.35delG is the most frequently recessively inherited mutation reported in nonsyndromic hearing loss, which is predicted to result in loss of functional Cx26 due to frameshift and premature termination codon at the early part of the protein. The development of the epidermis is not affected in the case of homozygous c.35delG patients.

The autosomal dominant inherited from *GJB2* mutations associated with hearing loss has been reported in both nonsyndromic and syndromic hearing loss patients.

The heterozygous c.32G>A mutation has been reported before in a Caucasian patient with KID syndrome (DOI: 10.1016/j.bbrc.2010.03.098).

There is no family history of hearing loss, suggesting either the low penetrance of this mutation or it being a de novo mutation only present in the affected child. Therefore, the combination of the 2 identified mutations leads to a severe phenotype when they are found in affected individuals as seen in our patient.

The constellation of the clinical features in our patient and the presence in the genotype of the “dominant” mutation c.32G>A (p.Gly11Glu) which leads to KID syndrome are totally compatible with the clinical phenotype of “keratitis-hearing loss-ichthyosis syndrome.”

## 3. Treatment

Conservative measures to treat the knee contractures were the first applied, which consisted of prone posturing with the hips and knees in extension and gentle passive manual elongation of the hamstrings to prevent permanent shortening. Active exercises to increase the motor strength of the quadriceps and the use of a night cast that holds the knee in extension and the ankle at neutral position have been tried. Crawling was not allowed, and other modalities of locomotion were offered.

Knee extension was assessed with the foot dorsiflexed and plantar flexed to estimate the contribution made by the gastrocnemius and with the hip abducted and adducted to assess the part being played by the gracilis. Most common muscles that are likely to be tight are the semitendinosus, gracilis, and biceps. When the quadriceps is strong, and flexion deformity is not greater than 40°, hamstring release is usually sufficient to provide correction and to restore muscle balance.

**Figure 2:**
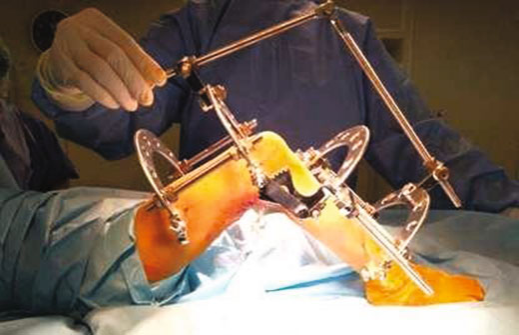
Extension of the hamstring tendons and installation of the external distraction apparatus through the application of Volkov- Oganesyan. A special ellipsoid hinge, simultaneously with the extension of the tibia, slightly shifts the anterior articular surface of the tibia, thereby preventing the formation of a posterior subluxation.

**Figure 3:**
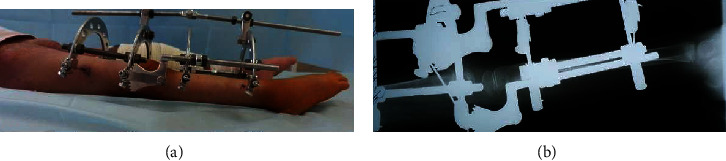
(a, b) Correction of knee joint deformation was achieved (a). Full extension of the knees has been achieved. Note that the lateral radiograph showed the full correction of the knee contractures (b).

Unfortunately, the response to the aforementioned procedures was not convincing enough to rely on. Therefore, we referred to a Volkov- Oganesyan apparatus, as it is specially organized for the correction of knee contractures (Figure 2). In the apparatus, there is a special ellipsoid hinge, which, simultaneously with the extension of the tibia, slightly shifts the anterior articular surface of the tibia, thereby preventing the formation of a posterior subluxation. When using the device, it was important to align the knee joints with the center of rotation of the knee. On the 5th day after the operation, gradual correction of contracture began, 5-8° degrees per day has been achieved. The full extension of the knee was satisfactory after 14 days (Figures 3(a) and 3(b)). Afterwards, a stabilization period was carried out in the apparatus. The second stage of surgical treatment was the removal of the frame from the lower limb and subcutaneous extension of the Achilles, with correction of foot deformation. Fixation in a cast lasted for 2 months, followed by the application of the knee-ankle-foot orthoses. After 6 months, a similar intervention was carried out on the opposite lower limb by the same applied methodology.

**Figure 4:**
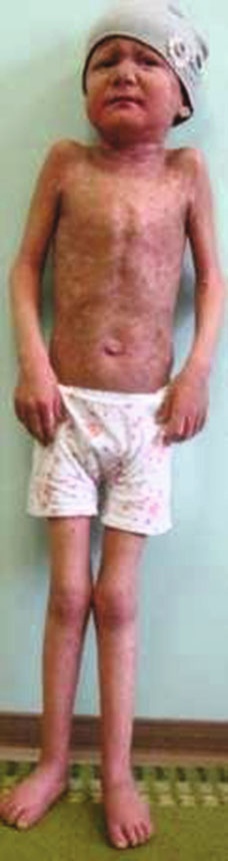
The child regained her locomotor capability through standing and walking. She started to walk with the support of the hand with the fixation of the lower extremities in the knee-ankle-foot orthoses. The mobility of the knee joints approached to somehow convincing results. Full extension, feet in the middle position, and with support of the entire plantar surface of the feet have been approached successfully.

Orthopedic procedures were complicated because of secondary infections associated with skin problems due to the pathological nature of the skin in KID syndrome patients. The result of treatment, after 1 year from the beginning, showed dramatic improvement and total control over infections. The child regained her locomotor capability through standing and walking. She started to walk with the support of the hand with the fixation of the lower extremities in the knee-ankle-foot orthoses. The mobility of the knee joints approached to somehow convincing results. Full extension, feet in the middle position, and with support of the entire plantar surface of the feet have been approached successfully (Figure 4). Currently, rehabilitation to enhance movements along the knees and ankle joints has been carried out. We recommended avoidance of increasing the flexion of the knee joint more than 90° and to continue the constant long-term wearing of orthoses to ensure the prevention of possible recurrence of contractures during the growth of the child.

## 4. Discussion

Keratitis-ichthyosis-deafness (KID) syndrome is characterized by congenital ichthyosis, but nevertheless, during the first weeks of life, there might be widespread erythroderma with desquamation. This is followed by the development of hyperkeratotic plaques, often localized on the face and limbs. Plantar and palmar hyperkeratosis is almost invariable, and it has a stippled and dotted pattern. The nails are variably affected and the hair is scanty and brittle. Hair histology might resemble that seen in trichothiodystrophy with bright and dark bands [1, 2, 6].

Previous studies described a broad spectrum of associated anomalies/deformities in patients with KID syndrome. Senter et al. [12] described a 13-year-old boy with atypical ichthyosiform erythroderma associated with congenital sensorineural deafness. The additional features were vascularization of the corneas, dystrophic nails, abnormal teeth, postnatal growth deficiency, and alopecia totalis. The skin lesions consisted of a form of lamellar ichthyosis with hyperkeratosis and acanthosis. Various forms of contracture of the foot were seen, and absent ankle jerks and nerve conduction studies indicative of a peripheral neuropathy have been described [1–6]. Dandy-Walker malformation as additional pathology and chronic lip fissures and gingival hyperemia were described in two different reports [13, 14]. Genetic and clinical heterogeneity has been described [15]. Desmons et al. [16] reported 3 sibs with ichthyosiform erythroderma and deafness. The parents were first cousins. There were no corneal abnormalities. Clouston [8] demonstrated mutations in the connexin 26 (GJB2) gene. Van Geel et al. [17] described another syndrome, called hystrix-like ichthyosis-deafness (HID) syndrome through specific missense mutation in connexin 26 (GJB2), which strongly resembles the KID syndrome. They assumed that HID and KID disorders are distinguished mainly on the basis of electron microscopic findings and hypothesized that KID and HID syndromes may be genetically related.

Montgomery et al. [18] described a congenitally deaf white male with mild palmoplantar keratoderma, ichthyosiform scaling, follicular hyperkeratosis, and mild keratitis, features consistent with keratitis-ichthyosis-deafness syndrome. But the patient's major problem was severe, disfiguring, inflammatory dissecting folliculitis of the scalp, hidradenitis suppurativa, and cystic acne, features comprising the follicular occlusion triad. This unusual phenotype is associated with a novel heterozygous point mutation (C119T) in the gap junction beta2 gene that substitutes a valine for alanine at codon 40 (A40V) in the connexin 26 protein. The phenotype and the natural history of the disease plus the genotype of our current patient are different.

## 5. In Summary

We presented a girl with phenotypic/genotypic characterization consistent with the diagnosis of KID syndrome. Fixed flexion deformity of the knees was a major malformation which hindered dramatically the locomotor system. Flexion deformity of the knees is not a common deformity encountered in children with KID syndrome. It was imperative to determine the primary site of the flexion deformity. Surgical procedures about the knees were performed with great caution.

In most cases with fixed flexion deformity of the knees, we usually start with correction of the flexion deformity of the hip. In cases with no hip deformity, or it has been corrected, it may still be profitable to correct deformities via preliminary passive stretching or serial plaster casts.

An Volkov- Oganesyan external hinged distraction device was applied through using transosseous pins and adjustable attachments. The overall success rate in our current patient was assessed via walking capacity, range of motion (ROM), and joint space width (JSW). The outcome of the application of external hinged distraction revealed that our results showed remarkable clinical improvement with regard to resuming standing and walking capabilities, with respect to knee motion, JSW, and tibiofemoral angle. As a result of treatment, we managed to eliminate contractures and restore the axis of the lower limbs, and the child began to walk.
